# Retraction Note: Prognostic value of heart failure in hemodialysis-dependent end-stage renal disease patients with myocardial fibrosis quantification by extracellular volume on cardiac magnetic resonance imaging

**DOI:** 10.1186/s12872-020-01688-7

**Published:** 2020-09-15

**Authors:** Hua-yan Xu, Zhi-gang Yang, Yi Zhang, Wan-lin Peng, Chun-chao Xia, Zhen-lin Li, Yong He, Rong Xu, Li Rao, Ying Peng, Yu-ming Li, Hong-ling Gao, Ying-kun Guo

**Affiliations:** 1 Department of Radiology, West China Second University Hospital, Sichuan University, 20# South Renmin Road, Chengdu, 610041 Sichuan China; 2 Key Laboratory of Obstetric & Gynecologic and Pediatric Diseases and Birth Defects of Ministry of Education, Sichuan University, 20# South Renmin Road, Chengdu, 610041 Sichuan China; 3 Department of Radiology, National Key Laboratory of Biotherapy, West China Hospital, Sichuan University, 37# Guo Xue Xiang, Chengdu, 610041 Sichuan China; 4 Department of Cardiology, West China Hospital, Sichuan University, 37# Guo Xue Xiang, Chengdu, 610041 China

**Retraction to: BMC Cardiovasc Disord 20, 12 (2020)**

**https://doi.org/10.1186/s12872-019-01313-2**

This article [1] is being retracted at the request of the authors. After publication, readers have raised concerns that gadolinium-based contrast agents (GBCAs) have been linked to the development of nephrogenic systemic fibrosis (NSF) in patients with severe renal insufficiency [2], such as the patients in end-stage renal disease (ESRD) who were enrolled in the current study. Most national guidelines advise that use of GBCAs in at-risk patients should only be considered for diagnostic purposes providing all alternative imaging modalities have been utilized. Moreover, the GBCA used in this study (Gadodiamide) is contraindicated in patients with severe kidney disease [3]. Unfortunately, the authors were unaware of these guidelines when the study was designed and ethics approval requested.

To ensure the safety of the patient and minimise risk of developing NSF, the researchers decreased the contrast dose given to patients and the patients underwent complete hemodialysis within 2 h of the imaging using the GBCA. The authors emphasize that the patients were closely monitored for side effects and that there was no decrease in any of the patients’ renal function after the scan, nor after their follow-up.

However, as the study contravenes internationally accepted guidelines, the authors would like to retract the article to avoid future use of GBCA in patients with ESRD without sufficient justification.

The authors have stated that they agree to this retraction.
